# Complications of lumbar disc herniations following trans-sacral epiduroscopic lumbar decompression: a single-center, retrospective study

**DOI:** 10.1186/s13018-017-0691-z

**Published:** 2017-12-04

**Authors:** Seung-Kook Kim, Su-Chan Lee, Seung-Woo Park, Eun-Sang Kim

**Affiliations:** 1 Department of Spine Center, Himchan Hospital, 118 Yongdam-ro, Yunsoo-gu, Incheon, 21927 South Korea; 2 Department of Neurosurgery, College of Medicine, Kangwon National University, Chuncheon, South Korea; 3 Joint and Arthritis Research, Orthopedic Surgery, Himchan Hospital, Seoul, South Korea; 4 Department of Neurosurgery, Spine center, Samsung Medical Center, Sungkyunkwan University School of Medicine, Seoul, South Korea

**Keywords:** Complication, Lumbar disc herniation, Epiduroscopic, Laser decompression, Minimally invasive spine surgery, Laser spine surgery, Endoscopic spine surgery, Trans-sacral epiduroscopic lumbar decompression

## Abstract

**Background:**

Trans-sacral epiduroscopic lumbar decompression (SELD) is an emerging procedure for the treatment of lumbar disc herniation (LDH), with favorable outcomes having been reported. However, the complications associated with SELD have not been comprehensively evaluated to date. Therefore, the aim of our study was to describe the incidence rate, types, and characteristics of complications following SELD and management outcomes.

**Methods:**

Retrospective analysis of the surgical and clinical outcomes for 127 patients (average age, 42.2 ± 15.2 years) who underwent SELD for LDH at L2-3, L3-4, L4-5, and/or L5-S1, performed by a single experienced spine surgeon at a single center, between January 2015 and April 2017, was conducted.

**Results:**

All procedures were successful, with a mean follow-up of 12.3 ± 2.3 months. Complications were identified in 8 patients (6.3%), including 3 cases of incomplete decompression (2.4%), 2 cases of recurrent disc herniation (1.6%), and one case each of hematoma, dural tearing, and subchondral osteonecrosis (0.8%). Among these cases with complications, only 2 cases with incomplete decompression and one case with recurrent LDH did not improve with conservative treatment and required re-operation using an open approach. The rate of complications decreased from 12.6% when considering only the first 50 cases to 2.6% for cases 51–127.

**Conclusions:**

Incomplete decompression, recurrent herniation, epidural hematoma, dural tear, and subchondral osteonecrosis were identified as complications of SELD, although the overall rate of complications was low. Practice with the procedure and careful patient selection can lower the risk of complications.

## Background

Lumbar intervertebral disc herniation (LDH) is a clinically symptomatic condition caused by compression of spinal nerve root by protruded disc material. The main symptoms of LDH are low back pain and radiating leg pain [1]. Treatment for LDH can be classified into microscopic open lumbar microdiscectomy (OLM) or a non-invasive pain-relieving procedure. OLM has been regarded as the standard treatment for LDH for many years, dating back to 1934 when Mixter and Barr reported partial laminectomy and disc removal for the treatment of LDH [2, 3]. However, this is an invasive procedure which requires general anesthesia, skin incision, and bone removal [3]. Owing to further exploration of the technique of percutaneous trans-sacral pain procedure and the development of video and surgical instruments, trans-sacral techniques have come to be widely used for the treatment of various spinal pain conditions. Trans-sacral epiduroscopic lumbar decompression (SELD) is an emerging field in research on non-invasive pain-relieving procedures for the spine. The underlying principle of SELD is to achieve sufficient decompression via laser vaporization of the nucleus pulposus, accessed through the sacral hiatus [4, 5]. This technology has a unique advantage for the treatment of centrally located disc herniations and a LDH at the level of L5-S1. SELD is particularly useful for the treatment of a LDH at L5-S1, which is easy to access from the sacral hiatus because of the short distance from sacral hiatus to the L5-S1 intervertebral disc. By comparison, accessing a centrally located disc herniation is difficult using a traditional open approach due to the need to interrupt the thecal sac and nerve root. As such, spine surgeons are increasingly interested in the application of SELD in these cases as the technique provides a similar procedural pathway as percutaneous epidural neuroplasty (PEN), as well as relying on the same anatomical references [6]. Although the use of SELD is increasing, the types of complications, as well as the rate of these complications, have not been clearly established. Therefore, even though some studies have reported favorable outcomes using SELD, because of the lack of outcome data, SELD is not considered as the first approach for the treatment of LDH and remains to be approved in some countries. Therefore, the aim of our study was to retrospectively evaluate the types, incidence, and characteristics of the complications of SELD performed for the treatment of LDH and to identify possible appropriate countermeasures to lower the risk for identified complications. To our knowledge, this is the first study to evaluate the risks associated with SELD, information that will influence the ongoing development of SELD technology and methods, as well as support further exploration of the breadth of its application to the treatment of LDH.

## Method

This study was approved by the Kangwon National University Institutional Review Board (KNU2017-08-002). We conducted a retrospective analysis of patients treated for LDH using SELD, performed by a single trained spine surgeon (S. Kim), between January 2015 and April 2017, at the Himchan Hospital, Incheon, Korea. Patients were screened using the following inclusion criteria: LDH located at L2-3, L3-4, L4-5, and/or L5-S1, confirmed by magnetic resonance (MR) imaging; main signs and symptoms of discogenic in nature and related to the LDH identified on MR images; and persistence of back and leg pain after 2 weeks of conservative treatment. Patients with a foraminal and extraforaminal LDH, tumor or infection, and with LDH combined with lumbar segmental instability were excluded. After screening, 128 consecutive patients were included in our analysis. The following information was extracted from the electrical medical records (EMR) for analysis: age, disease duration, sex, main disc lesion, type of herniation, and disc consistency. The following clinical outcomes were assessed: Visual Analogue Scale (VAS) score for leg pain, Oswentry Disability Index (ODI), patients’ rating of satisfaction rate, using a questionnaire from our institution (Joint and Arthritis Research Himchan Hospital), which is based on the modified Macnab criteria. Data were compared across three time points of measurement: preoperative, immediate postoperative, and at final follow-up (1 year). Patients underwent pre- and postoperative radiographic and MR imaging of the lumbar spine.

### Procedural techniques

The procedure was performed in the prone position by Dr. S. Kim. The entry point in the skin was generally at the midline of the sacral hiatus for epidural administration. After local infiltration of the entry point with an anesthetic, a 22-gauge needle was introduced under radiographic guidance. The final target point of the epiduroscopic catheter was the posterior line of the vertebral body, visible on lateral view. Next, an epidurography was performed using contrast media to confirm the location of the traversing and exiting components of the spinal root and the LDH. The following steps were then performed: a small insertion point was made in the skin at the entry point; a tapered cannula was administered through sacral hiatus; and, lastly, a laser and camera-equipped epiduroscope (Meta-bio-med; i-polphin®, Korea) was inserted below the disc along the cannula, and the elevated pathological nucleus and ruptured fragment of the LDH visualized. The pathological disc was decompressed using a holmium:yttrium aluminum garnet (Holm:YAG) laser. After the herniated fragment was ablated, the epiduroscope was removed and a sterile dressing applied, without suture. A PEN was performed, using 10 ml of a mixture of 0.5% bupivacaine and 1500 IU hyaluronidase and administered using an epiduroscopic catheter after the nerve was sufficiently decompressed, which indicated a favorable patency of contrast medium following the nerve root and mobility of the epiduroscope.

All patients received a 3-day course of antibiotics, non-steroidal anti-inflammatory drug, and H2 blocker. Patients were instructed to walk and begin back exercises 2 h after the procedure. Patients were followed up postoperatively through the outpatient clinic at 2 weeks; one, 3 and 6 months; and at 1 year post-surgery. For patients with residual symptoms, a 4-week course of conservative treatment was prescribed. Repeat MR imaging was performed at the 2-week follow-up visit, with visualization of the nerve root over its course through the spinal canal and intervertebral foramen being radiologic evidence of complete decompression.

### Statistics

All EMR, preoperative and postoperative imaging, and clinical data were collected and analyzed. The complication rate was calculated for the first 50 cases and for the remaining 77 cases and compared to evaluate possible learning effects of the SELD technique on outcomes. In the same way, the complication rate for L4-5 and L5-S1 discs were compared. One patient was lost to follow-up and removed from the analysis. Between-group differences were evaluated using Levene’s test. When the test rejected the hypothesis of equal variance between the two groups, between-group differences were evaluated using a one-way analysis of variance (ANOVA), with Bonferroni correction for multiple comparisons. The Kruskal-Wallis test and chi-squared test were used for non-normally distributed data to evaluate between-group difference. Statistically significance was set at a *p* value < 0.05. All analyses were performed using SPSS 20.0 software (SPSS Inc., Chicago, IL, USA).

## Results

Of the 128 patients enrolled into our study, one patient was lost to follow-up and removed from the planned analysis. The remaining patients received a total of 127 procedures. The study group included 83 men (65.4%) and 44 women (34.6%), with a mean overall age of 42.2 years (range, 22 to 65 years) and a mean duration of symptoms of 12.3 months (range, 12 to 14 months). General information and clinical data for patients forming the study group are provided in Table 1. Clinical outcomes were summarized in Table 2. Pain and life qualities significantly improved after 1 year follow-up.

**Table 1:** Relevant characteristics of the study group

	Mean	*N* (%)
Population		127 (100)
Age (years)	42.2 ± 15.2	
Duration of low back pain (months)	12.3 ± 2.3	
Sex		83 (65.4)
Male		44 (34.6)
Main disc lesion		
L2-3		2 (1.6)
L3-4		5 (3.9)
L4-5		50 (39.3)
L5-S1		70 (55.1)
Type of herniation		
Central		73 (57.5)
Paracentral		54 (42.5)
Disc type		
Hard (calcification)		48 (37.8)
Soft		79 (62.2)

**Table 2:** Clinical outcomes of the study group

	Preoperative	Postoperative	*p* value
Visual Analogue Scale back pain	5.32 ± 1.5	1.32 ± 1.0	0.001
Visual Analogue Scale leg pain	6.13 ± 1.1	1.27 ± 1.2	0.001
Oswentry Disability Index (%)	65.15 ± 1.0	12.1 ± 1.4	0.001

**Fig. 1:**
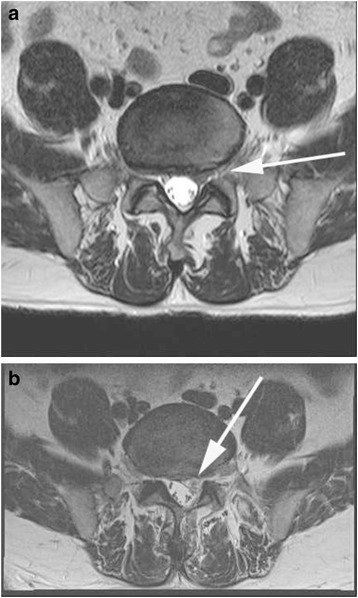
**a** A T2WI magnetic resonance (MR) image showing an incomplete decompression, with protrusion of the L5-S1 disc compressing the S1 nerve root. **b** Postoperative MR image showing the postoperative microscopic discectomy state, with the disc removed and nerve released

Complications were identified in 8/127 patients (6.3%) and included incomplete decompression, recurrent herniation, epidural hematoma, dural tear, and subchondral osteonecrosis. The incidence rate of complications was 55.1% at L5-S1 compared to 39.3% at L4-L5 with statistical significance (*p* = 0.048). The rate of complication at L4-L5 and L5-S1 was greater than that at L3-4 (3.9%) and L2-3 (1.6%), this difference being non-significant (*p* = 0.75). A centrally located disc herniation (37.8%) and soft disc consistency (62.2%) were common features of the LDHs. The distribution of complications is provided in Table 3. Moreover, among the first 50 patients who underwent SELD, related complications were identified in 6 patients (12%), with this rate subsequently decreasing to 2 patients (2.6%) for cases 51 through 127. Over the 127 patients, 3 patients (2.4%) presented with residual symptoms, without a pain-free interval. In these cases, residual symptoms included back pain and radiating leg pain that were similar to preoperative intensity, caused by an incomplete decompression, confirmed by MR imaging (Fig. 1a). In these 3 cases, disc degeneration was combined with recess stenosis and calcification. During the procedure, the nerve root was pushed posteriorly by the bulging annulus fibrosus and was adherent to the ligamentum flavum, with inflammatory vasculature wrapped round the nerve root. Laser stimulation of the nerve root during the procedure caused paresthesia and, consequently, sufficient decompression could not be achieved. Among these 3 cases, 2 patients underwent open laminectomy and removal of the disc and ligamentum flavum at 2 and 7 days after the initial procedure (Fig. 1b). For the other patient, symptoms and the degree of residual compression gradually decreased after 1–4 weeks of conservative treatment.

**Table 3:** Distribution of complications

	All cases	Cases 1–50	Cases 51–127	L4-5 (50)	L5-S1 (70)
Total	8 (6.3%)	6 (12%)	2 (2.6%)	5 (10%)	3 (4.3%)
Incomplete decompression	3 (2.4%)	2 (4%)	1 (1.3%)	2 (4%)	1 (1.4%)
Recurrent herniation	2 (1.6%)	1 (2%)	1 (1.3%)	1 (2%)	1 (1.4%)
Epidural hematoma	1 (0.8%)	1 (2%)	0	1 (2%)	0
Dural tear	1 (0.8%)	1 (2%)	0	1 (2%)	0
Subchondral osteonecrosis	1 (0.8%)	1 (2%)		0	1 (1.4%)
*p* value		0.041		0.048

**Fig. 2:**
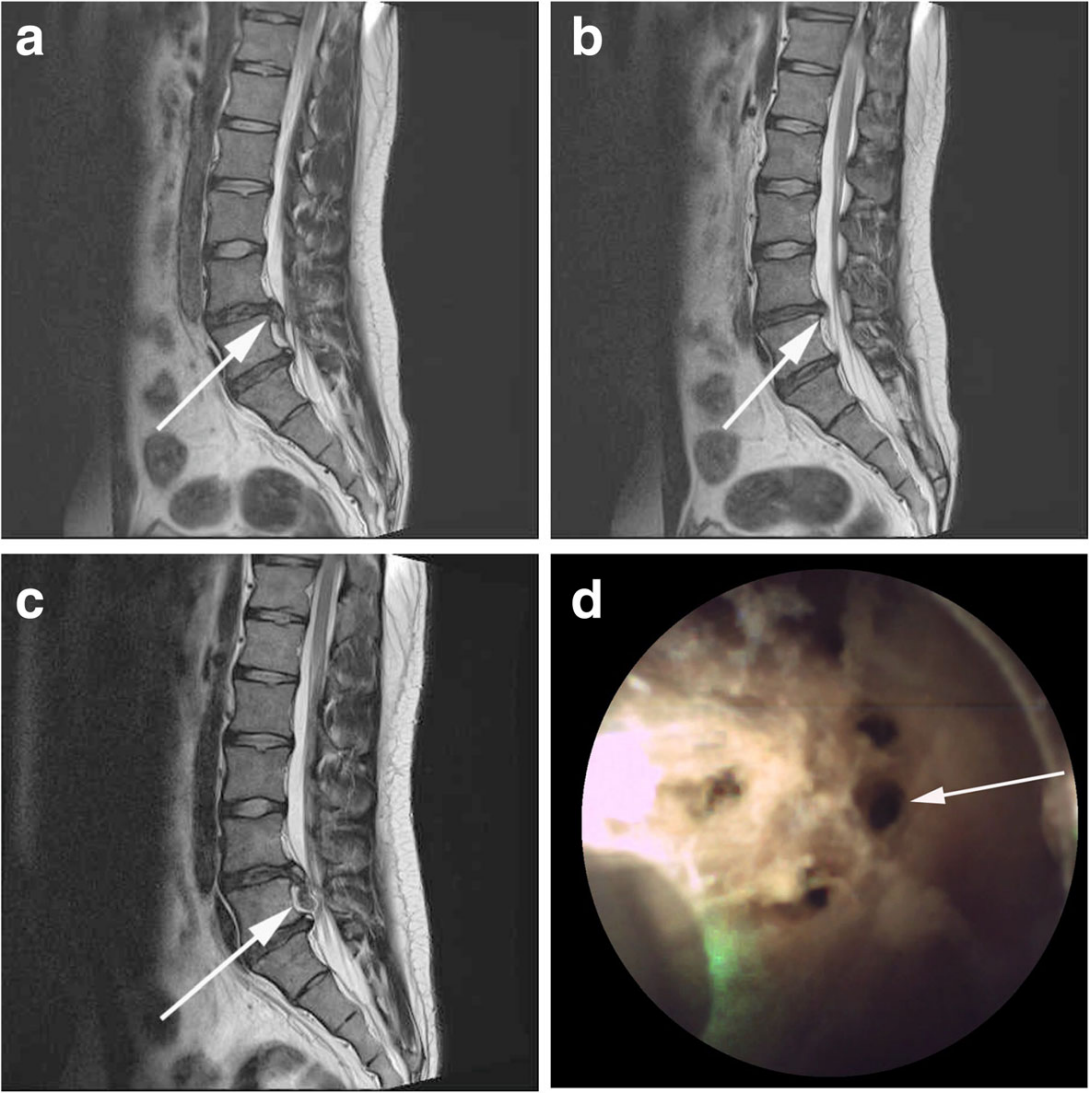
**a** Preoperative sagittal MR image showing a ruptured L4-5 disc with downward migration. **b** Sagittal MR image obtained 2 weeks postoperatively, showing decompression of the disc. **c** Recurrence of the disc rupture. **d** Epiduroscopic image showing multiple disc ablation holes. These multiple holes were likely to be the origin of the recurrence of the disc herniation

Over the follow-up period, 2 cases of recurrent lumbar disc herniation (RLDH) were recorded (1.6%). One of these cases was a 36-year-old female who complained of ipsilateral radicular pain after the procedure (Fig. 2a). The patient required multiple laser ablations of the ruptured disc to achieve sufficient decompression (Fig. 2b), which was confirmed on postoperative MR imaging and was associated with symptom improvement. However, at 6 months post-surgery, the patient complained of recurrent symptoms after heavy lifting and a RLDH was confirmed on MR imaging (Fig. 2c). On the endoscopic view, multiple vaporization holes for decompression were observable, which likely contributed to the RLDH (Fig. 2d). For the other patient, a RLDH was identified at 8 months after the procedure; however, symptoms improved in this patient after conservative treatment.

**Fig. 3:**
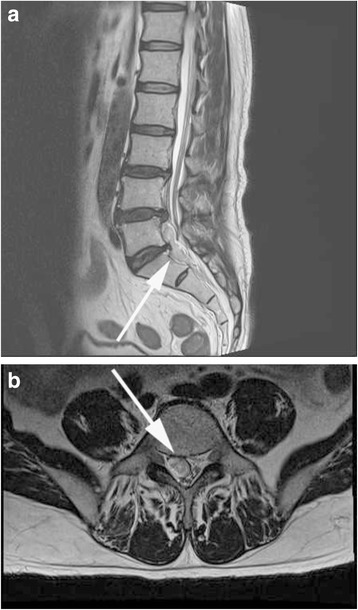
**a** T2WI sagittal plane MR image showing an epidural hematoma extending from L4 to S1 level. **b** T2WI axial MR image showing the epidural hematoma

**Fig. 4:**
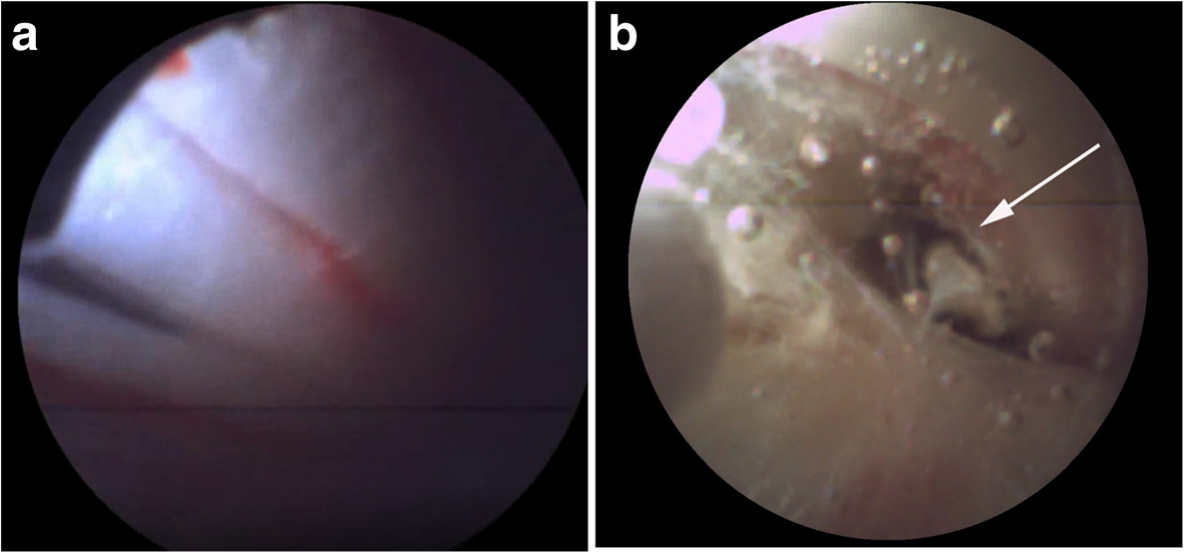
**a** Epiduroscopic image obtained when the intradural catheter was inserted, showing the cauda equina. **b** Epiduroscopic image showing the central tearing point in the dura

**Fig. 5:**
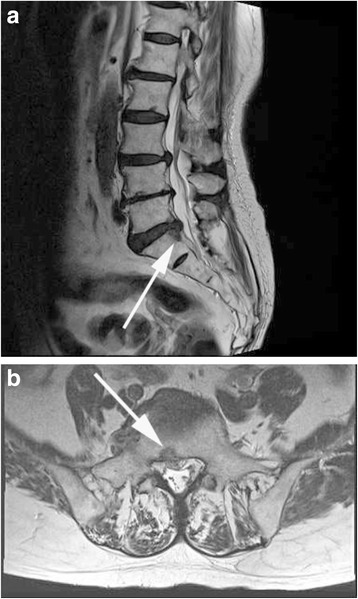
**a** T2WI sagittal image showing a low-intensity signal due to subchondral osteonecrosis. **b** T2WI axial image showing subchondral osteonecrosis at the laser ablation site

The following complications were also identified in our study group. A 48-year-old female complained of back pain 6 h after the procedure due to the development of an epidural hematoma (1 case, 0.8%). On sagittal (Fig. 3a) and axial (Fig. 3b) MR imaging, the hematoma was found to be compressing the thecal sac, from L4 to S1. The patient recovered with 2–4 weeks of rehabilitative exercises. A dural tear was identified in one case (0.8%), a 52-year-old male. Tearing occurred during insertion of the epiduroscope into the thecal sac. Immediately after we identified the cauda equine, cerebrospinal fluid gushed out (Fig. 4a). After careful retraction of the epiduroscope, the dural tear could be observed at the sacral level (Fig. 4b). Subchondral osteonecrosis was also identified in one patient (0.8%), a 62-year-old female. The subchondral osteonecrosis was identified on the regular follow-up MR image obtained at 2 weeks after the procedure. In this case, disc decompression was performed at a Holm:YAG laser intensity of 0.7 J (W = kgf m/s), applied at a frequency of 10 Hz for 120 s. MR imaging revealed a wedge-shaped low-intensity signal on T2WI sagittal (Fig. 5a) and axial (Fig. 5b) image.

The overall complication rate for the first 50 patients was 12% and included incomplete decompression (4%), RLDH (2%), epidural hematoma (2%), dural tear (2%), and subchondral osteonecrosis (2%). The complication rate decreased to 2.6% (2 patients) for the subsequent 77 cases, including: incomplete decompression (1.3%) and RLDH (1.3%). Specifically, for the first 50 patients and last 50 patients, the complication rate decreased from 12.0% to 2.6% (*p* = 0.041). Furthermore, the rate of complication was greater for L4-5 LDHs (10%) than for L5-S1 LDHs (4.3%, *p* = 0.048).

## Discussion

SELD has gained popularity for the treatment of LDH, with both spine surgeons and patients, due to its non-invasive nature, short postoperative hospital stay, use of local anesthesia, and high level of effectiveness. The use of SELD for the treatment of LDH has been widely validated and has been shown to produce favorable outcomes even in cases with lumbar spinal stenosis [7]. However, the risk and type of complications associated with SELD have not been comprehensively examined, compared to those associated with laser decompression via a trans-foraminal approach [8, 9]. Due to differences in the entry point and skill required for the procedure, the complications are likely to be different for trans-foraminal and trans-sacral approach epiduroscopic procedures. Over 164 cases of laser discectomies, Tonami et al. and Ohnmeiss et al. reported the following overall rates of complications: 12 cases (7.3%) of postoperative radiating dysesthesia, which progressively improved in 5 cases; and 2 cases of reflex sympathetic dysfunction [9, 10]. Based on these results, the complication rate of laser decompression (7.3%) is deemed to be lower than the rate for endoscopic (10.9%) and micro (19.5%) surgery [11]. In their case series of 197 laser decompressions, Erbas et al. reported a complication rate of 13.7%, with the majority of these being incomplete decompression (12.7%) and subchondral osteonecrosis (1%) [12]. Although these previous studies included cases for which a trans-sacral approach was used, these cases were not evaluated separately. Our study described the types, incidence, and characteristics of the complications following SELD in detail.

Incomplete decompression always results in persistent postoperative radiating pain, without a pain-free interval after surgery [8, 13]. Zhao et al. [14] indicated that herniations with stenosis (degeneration) and high-grade migration of the nucleus are associated with the highest rate of incomplete decompression. As the basic principle of SELD is the vaporization of nucleus pulposus, we propose that SELD would be most appropriate for the treatment of a recent disc herniation without degeneration. The presence of a high-grade migration of the disc, especially in the upward direction, significantly decreases the mobility of the epiduroscope, increasing the difficulty of the decompression. Therefore, preoperative identification of degeneration findings and migration should lead to careful consideration of the possibility of achieving sufficient decompression.

RLDH has been reported following various surgical approaches to lumbar discectomy [15–17]. Suk et al. [18] defined a RLDH as a disc herniation at the same level, following a pain-free interval > 6 months after surgery, regardless of whether the recurring pain is ipsilateral or contralateral to the initial side. Known risk factors for RLDH include sex, smoking, body mass index, and diabetes [19]. The primary cause of RLDH is continued degeneration of the surgically treated disc. In our case series, one patient, a 36-year-old female, required re-operation due to a RLDH. This patient had undergone multiple disc ablations to achieve sufficient decompression, which likely led to the RLDH (Fig. 2d). Thus, we propose that multiple vaporization holes and exogenic action may be precipitating causes of RLDHs. Kim and Park [20] compared outcomes for patients treated for LDH, *with* and *without* the use of annular sealing, reporting a significantly lower rate of recurrence (5.5%) with annular sealing than without (13.5%). In our case series, we did not perform either annular sealing or annuloplasty. With regard to decompression, performing multiple ablations can be an effective technique to achieve sufficient decompression for favorable outcomes, although this approach does increase the risk for RLDH. To reduce this risk, patients should be instructed to perform back exercises and avoid heavy lifting for as long as possible.

A previous study has reported persistent paresthesia as a symptom of epidural hematoma after trans-foraminal percutaneous laser lumbar discectomy [8]. In their retrospective analysis of 658 cases of laser lumbar discectomy, performed across nine centers, Mayer et al. [21] reported 4 cases (0.6%) of vascular injury causing a hematoma. Of note, inflammatory bleeding caused by an increase in blood pressure during injury to the annulus by the Holm:YAG laser may blur the surgical field and influence patients’ symptoms after surgery. In our case, as the patient’s symptoms were benign in nature and recovered, albeit slowly, we did not consider removal of the hematoma as a necessary therapeutic option. Achievement of effective hemostasis and a clean procedure field are important in reducing the risk of vascular injury and hematoma formation. Gentle and accurate manipulation and control of blood pressure are further necessary to lower the risk of hematoma.

Dural tears is a well-known complication of lumbar spinal surgery [22]. As SELD does not use a knife or Kerrison rongeur, the possibility of dura tearing is extremely low. Moreover, as the diameter of the epiduroscope is small, any tearing also tends to be small, often being present without overt symptomology and, therefore, not requiring supine rest with a pressure dressing. Any patient with a serious tear, which can cause uncontrolled cerebrospinal fluid leakage and even nerve root herniation will require conversion to open surgery for repair [23–26].

Subchondral osteonecrosis is an abnormal signal intensity identified in bone located directly below or adjacent to the region of the intervertebral disc issue treated with laser energy [9]. Tonami et al. [9] reported 4 cases of subchondral osteonecrosis in their series of 189 cases (2.2%) of percutaneous laser decompression of lumbar discs. Laser-induced osteonecrosis can result from various causes. Foremost, bone death can be induced by use of excessively high temperature and indirectly from pressure changes associated with injury to the subchondral bone in a manner similar to the occurrence of osteochondritis from a photoacoustic insult [27, 28]. The clinical significance of subchondral osteonecrosis has not yet been studied. In the one case in our case series, the patient complained only of lumbar back pain, with symptoms recovering following 2–4 weeks of rehabilitation therapy.

The efficacy of epidural neural decompression with laser has been recently introduced and is being considered as a new treatment modality for various conditions, including LDH, spinal stenosis, post spinal surgery syndrome, and chronic persisting lower back pain despite continuous conservative treatment [4]. SELD showed superior effectiveness above both caudal epidural injections and physiotherapy [29, 30]. Furthermore, continuous laser exposure has been reported to cause dural damage [31]. Postoperative headache, pain at the insertion site, pain and infection have been reported with SELD [7]. Our study suggests postoperative headache and back pain are likely to result from dural tearing, incomplete decompression, RLDHs, and subchondral osteonecrosis.

Of note is the steep decrease in the rate of complication after the first 50 SELD cases. These findings are consistent with a relatively fast learning curve for SELD. The higher complication rate for procedures at L4-5 than at L5-S1 reflects the fact that the L5-S1 disc space must be traversed to reach the L4-L5 disc. The lumbo-sacral angle and protruded L5-S1 disc may hinder the mobility of the epiduroscope, which would affect the accuracy of approach of the target point and of the subsequent disc decompression. Based on these findings, SELD might be more suitable for the treatment of L5-S1 LDH, which is closer than L4-5 to the sacral hiatus.

As with any study, there are some limitations to our study which need to be acknowledged. Foremost, this is a retrospective design, with no randomization or control group. Therefore, there is a possibility that the rate of complication was underestimated and patient selection influenced complication of this procedure. Second, our sample size was relatively small and with a short follow-up period. Despite these limitations, we believe that this study is clinically important and provides a comprehensive analysis of complications associated with SELD, as well as provides information on the effects of surgeon’s learning the technique on outcomes.

## Conclusion

SELD provides a basic treatment for LDH that is non-invasive and only requires local anesthesia and is associated with a low risk of complications. Complications typically result from improper selection of patients and non-skilled procedure. The risk of complication can be reduced by having surgeons train well on the technical performance of the procedure, appropriately selecting patients, and providing appropriate perioperative management.

## Abbreviations

EMR: Electrical medical records
LDH: Lumbar intervertebral disc herniation
MR: Magnetic resonance
OLM: Open lumbar microdiscectomy
RLDH: Recurrent lumbar disc herniation
SELD: Trans-sacral epiduroscopic lumbar decompression
